# Steroid Hormone Receptors: Links With Cell Cycle Machinery and Breast Cancer Progression

**DOI:** 10.3389/fonc.2021.620214

**Published:** 2021-03-12

**Authors:** Suryendu Saha, Samya Dey, Somsubhra Nath

**Affiliations:** Department of Basic and Translational Research, Saroj Gupta Cancer Centre and Research Institute, Kolkata, India

**Keywords:** steroid hormone receptors (SHRs), cell cycle, breast cancer, estrogen receptor (ER), progesterone receptor (PR), androgen receptor (AR)

## Abstract

Progression of cells through cell cycle consists of a series of events orchestrated in a regulated fashion. Such processes are influenced by cell cycle regulated expression of various proteins where multiple families of transcription factors take integral parts. Among these, the steroid hormone receptors (SHRs) represent a connection between the external hormone milieu and genes that control cellular proliferation. Therefore, understanding the molecular connection between the transcriptional role of steroid hormone receptors and cell cycle deserves importance in dissecting cellular proliferation in normal as well as malignant conditions. Deregulation of cell cycle promotes malignancies of various origins, including breast cancer. Indeed, SHR members play crucial role in breast cancer progression as well as management. This review focuses on SHR-driven cell cycle regulation and moving forward, attempts to discuss the role of SHR-driven crosstalk between cell cycle anomalies and breast cancer.

## Introduction

Cell cycle progression is a finely regulated process with several checks and balances ensuring that division and proliferation is a favored outcome. The fidelity of cell cycle is regulated by three vital checkpoints, governing the boundaries at G1/S, G2/M, and metaphase/anaphase transition (1). Execution of cell cycle is aided by timely functioning of these checkpoints and associated proteins, deregulation of which influences the occurrence of malignancy (2). For instance, checkpoint error during mitotic progression might result in generation of daughter cells with altered ploidy level (aneuploidy), a hallmark prevalent in almost 70% of solid human tumors (3). Thus, the cell cycle and cancer are found intertwined: cell cycle regulates cell proliferation, and cancer is a disease of unchecked cell proliferation.

As a measure of regulation, there are families of transcription factors which take charge in the timely expression of the cell cycle specific protein molecules. Among them, “steroid hormone receptors (SHRs)” represent an important group of transcription factors, having roles in cellular growth and proliferation (4). Members of this family act upon binding to a steroid hormone ligand. Importantly, evidences accumulated the involvement of SHRs in various cancers and showed their immense potential in targeted therapy (5). Indeed, two members of this family (Estrogen receptor/ER, and Progesterone receptor/PR) are well documented in breast tumor biology as well as they act as clinically established targets in breast cancer (6). Additionally, another member of the SHR family, androgen receptor (AR), is also gaining importance, specifically in the field of triple negative breast cancer (7). ER and PR (and also, AR) have verified functions in different phases of cell cycle. These cell cycle specific functions and mostly their alterations ought to be instrumental in bringing breast malignancy. With a brief section covering the structure and general functions of SHR, this review focuses on finding the SHR-driven cross-talk between cell cycle and breast cancer.

## Biology of Steroid Hormone Receptors (SHRs): Structure and Function

**Figure 1:**
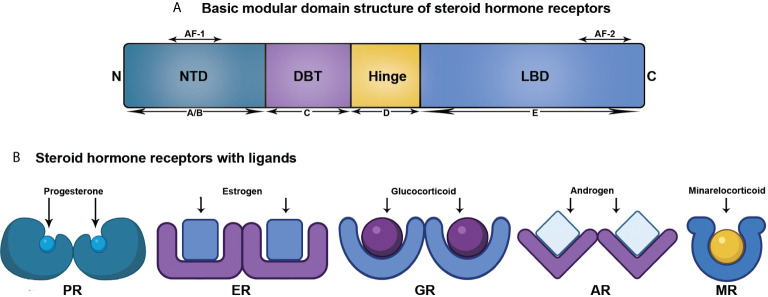
Structural organization of SHRs associated with ligand. **(A)** Linear representation of domains of SHRs. **(B)** SHRs and their corresponding ligand.

As per the present literatures, the members comprising the SHR family are ER, PR, AR, mineralocorticoid receptor (MR), and glucocorticoid receptor (GR) (8). Though this review centers around ER, PR, and AR, all the SHR members structurally comprise of five domains, as discussed below (**Figure 1**).

A/B: N-terminal domain (NTD): This is a highly variable domain and harbors a little similarity in terms of sequence and size among the SHR members. It also possesses at least one activator function-1 region (AF-1), that acts in a ligand-independent manner (9, 10). The NTD is the target for varied post-translational modifications with variable effects in driving or repressing transcription (11).

C: DNA binding domain (DBD): Among the domains of SHR, the centrally located and mostly preserved DBD contains two zinc finger motifs with each having four cysteine residues. An associated amphipathic helix and an amide loop trail each of these two motifs. Of them, the primary one comprises of the DNA-reading helix that communicates with the principal depression to establish base-explicit associations with DNA. The next motif that has the peptide loop to promote the dimerization of receptors by means of the distal or D box, attaches vaguely with DNA (11, 12).

D: Hinge Region: This region is a small, versatile, and the least conserved amino acid sequence connecting the DBD and LBD. Although the precise function of this domain is unclear, it acts as a site of post translational modifications (PTMs), associated with increased transcriptional activation. Moreover, a nuclear localization signal is also present within this domain (11, 12).

E: Ligand-binding domain (LBD): This domain exhibits a ligand-dependent activation function motif (AF-2), comprising of three helices (3, 4, and 12) present at the C-terminal end. It recruits several coregulators by means of changing conformation of helix 12, upon interacting with ligand. LBD is structurally preserved to a lesser extent and has 11–13 α-helices and four β-strands, organized into three layers in parallel, to create a hydrophobic pocket for ligand binding (LBP). The LBD of SHR shows a higher degree of identity at the upper part, however, the lower part containing LBP confers considerable variation across SHR members to facilitate the interaction of various cadres of ligands (11, 12).

### Classical Mode of SHR functions

In absence of a steroid hormone ligand, SHR remains sequestered by a chaperone, heat shock protein 90 (Hsp90) (4, 8). In the classical mode of SHR activation, after ligand docking, a ligand-specific conformational modification of the receptor happens, permitting its dissociation from restrictive Hsp90 complex, followed by auto-phosphorylation (4, 8, 13). These modifications trigger homo- or heterodimerization of SHR, followed by its nuclear translocation and subsequent binding to hormone response element (HRE) in a target gene. This, in turn, initiates the SHR-driven transcriptional regulation of a target gene (4, 8). The ligand-induced conformational modification of a SHR also facilitates its association/dissociation with coregulator complexes, during the course of transcription (4, 8, 12, 14).

In case of estrogen receptor (ER), there exists two isoforms, ER-α and ER-β, each of them translated from completely different genes with similar however not identical structure (15, 16). E2 (17β-estradiol) is the steroid hormone ligand that binds to ER, changing its conformation and activating ER-mediated transcription. E2-activated ERs homodimerize and function as transcription factors, upon interacting with estrogen response element (ERE) on a target gene (17). Likewise, there exists two isoforms of progesterone receptor (PR): PR-A is deleted for 164 amino acids at the N-terminal end, while PR-B refers to the entirely intact receptor. An additional isoform, PR-C is represented with a truncation in the DBD and is efficient in impeding the function of PR-B. Progesterone acts through PR-A and PR-B; however, the functional roles of PR-C remain to be characterized (18).

The classical or genomic androgen receptor (AR) signaling pathway involves the dissociation of chaperones from the androgen-activated AR, followed by its translocation to the nucleus. Dimerized AR attaches to androgen response element (ARE) within the genome, leading to AR-mediated transcription of the target genes. This is assisted by recruitment of several coregulators, resulting in the expression of androgen-regulated genes (19).

### Non-Classical Mode of SHR Function

Though the members of SHR family are mostly localized in nucleus (ER and PR) or cytoplasm (AR, MR, and GR), a distinct pool of them, approximately 5%, are located at the plasma membrane, including ER, PR, and AR (20, 21). This pool is responsible of extra-nuclear signaling cascades mediated by SHR and this defines the non-classical mode of SHR function. The membrane trafficking of these receptors is initiated by palmitoylation of an internal cysteine residue, which is driven by two palmitoylacyltransferase proteins, DHHC7 and DHHC21 (22, 23). Cav-1 transports the palmitoylated receptors to the caveolae rafts in the membrane. Indeed, Cav-1 also serves as a scaffold protein for different signaling molecule assembly, including G-protein-coupled receptors, protein kinase C, components of the MAPK pathway, and endothelial nitric oxide synthase (eNOS), to SHR. Although membrane-associated signaling pathways are extensively diverse for different steroid hormones, some of the common pathways include: ion fluxes (mostly calcium), secondary messengers such as inositol trisphosphate (IP_3_) and cyclic AMP (cAMP), activation of PKC and the extracellular signal regulated kinase–MAPK (ERK-MAPK), and PI3K-Akt pathways (20, 21, 24, 25). Membrane steroid receptors (mSRs) are also reported to mediate the quick non-genomic effects of steroid hormones through inhibition of adenylate cyclase (AC) and cyclic nucleotide (cAMP) production and activation of MAPK (26).

Membrane-coupled ER, upon ligand binding, dimerizes which in turn, makes it interacting to G protein alpha and beta/gamma subunits. This results in various rapid signal transduction events including early kinase (e.g. SRC) activation (21). In endothelial cells, membrane localized caveole-bound ER can activate endothelial nitric oxide synthase (eNOS) through protein kinase-mediated phosphorylation (27). Binding of E2 to scaffolded ER-α in the caveole results in interaction with G_αi_ proteins and activation of the PI3k-Akt pathway that involves subsequent eNOs activity and rise in nitric oxide levels (28). Additionally, the presence of ER-β at the cell membrane suggests that it can mediate extranuclear actions through its association with steroid receptor coactivator (Src) (29). There is another ER variant (ER-α-36), acting at non-classical level, and is primarily localized in plasma membrane and cytosol. It expresses through alternative splicing, lacking both of the transactivation domains, as present in the classical ER-α. Upon binding to various ligands, it involves in rapid membrane-initiated mitogenic signaling pathways, such as MAPK/ERK, PI3K/Akt, and PKC-δ to regulate biological functions of cells (30).

Another integral membranous receptor is GPR30, which is coupled to Gαs in its inactive state and its activation causes heterotrimeric G proteins to induce adenylate cyclase, Src, and EGFR signaling (31, 32). Through mPR, progesterone alters signal transduction pathways by activating mitogen-activated protein kinases (MAPKs) and inhibiting adenylyl cyclase and cAMP formation. Upon progesterone binding, mPR involves in various signaling cascades, including stimulation of extracellular signal-regulated kinases 1/2 (ERK1/2orp42/44) or p38MAPKs, and stimulation of intracellular Ca2+ mobilization (33). E2-activated GPR30 can activate downstream adenylate cyclase, AKT, and MAPK signaling (34). Further, reduction of GPR30 expression is reported to stop growth stimulation of TNBC cells (that lack ER-α) by E2 (35, 36); conversely, different reports counsel that GPR30/GPER expression correlates with higher prognosis in ER-α positive carcinoma patient (36, 37).

The extranuclear activity of progesterone is channeled by membrane progesterone receptor (mPR) and also the membrane-bound progesterone receptor membrane component one (PGRMC1). In most of the cell models examined, progestin-bound mPR-α involves in alteration of second messenger pathways, by activating pertussis toxin-sensitive inhibitory G-proteins (38). The ligand-activated extranuclear PR can interact with c-Src, by means of a polyproline (PPD) domain within its N-terminal domain. This interaction triggers rapid activation of Src/Ras/Raf/MAPK and other downstream targets (39). In human myometrial cells, mPR-α can activate p38, a MAPK, that phosphorylates myosin light chain protein in these cells (40). Another signaling pathway, which is executed in Atlantic croaker oocytes through mPR-α, is the Akt/PI3 kinase pathway (41). Progestin induction of mPR-α can either increase (42) or decrease (40) the cAMP production through the involvement of two different G proteins. A number of studies suggest both mPR-α and PGRMC1 act in concert to initiate calcium mobilization from intracellular compartments, upon progestin binding (43, 44). PGRMC1 also contains binding sites for Src homology 2 (SH2) and Src homology 3(SH3) domain-containing proteins (45).

Studies on membrane-associated androgen receptor depicted that it can interact with AKT to activate the downstream signaling, upon androgen stimulation (46). A splice variant lacking NTD, AR45 is localized in plasma membrane that might involve in intracellular calcium signaling upon interacting with the membrane-associated Gαo and Gαq proteins (47).

### Integration of Non-Classical and Classical Mode of SHR Functions

**Figure 2:**
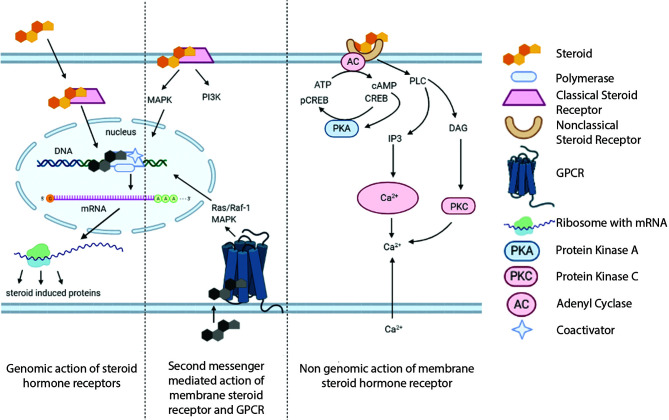
Convergence of genomic and nongenomic mechanisms of steroid hormone receptor action. Both of these pathways together can alter biological processes.

Importantly, these non-classical effects could integrate with classical mode of SHR action, in which the extranuclear signal pathways converge on SHR-driven transcriptional regulation. Apart from kinase activation and calcium signaling at the extranuclear compartment, rapid membrane-associated pathways could result in downstream genomic effects through regulating transcription factors and cofactors (**Figure 2**).

Several studies on knockout mice enhance the current understandings of the highly coupled genomic and nongenomic action of SHRs. As E2-dependent lipid biogenesis was decreased in a nuclear receptor knockout mice, it indicates extensive membrane ER signaling, resulting in phosphorylation and sequestration of the transcription factor SREBP1c at cytoplasm. This renders the critical lipid synthetic genes to remain inactive in the nucleus (48). Contrariwise, the mice expressing only nuclear ER that exhibited the membrane-initiated ERK-MAPK cascade by membranous ER-α, was extremely necessary in driving the recruitment of nuclear ER to the promoter and subsequent transactivation of pS2 gene (49). Another study discussed the role of AR in cell motility and invasiveness in TNBC, where the authors documented AR/Src assembly upon androgen induction (50). This complex triggers downstream non-classical signaling, that involves the recruitment of p85α, the regulatory subunit of PI3K and, FAK (Focal Adhesion Kinase). The activated PI3K phosphorylates Akt at Ser 473 residue, while autophosphorylation of FAK (on Tyr 397) results in Tyr 118 phosphorylation of paxillin. These two signaling cascades act in concert to promote migration and invasiveness of TNBC cells (50, 51). On a similar note, a membrane-localized splice variant of AR, AR8, is reported to promote the interaction of Src and AR with EGFR, upon EGF stimulation. Membrane-associated AR8 brings AR in close proximity of the plasma membrane to facilitate the formation of a dynamic signaling complex, with AR, AR8, Src, and EGFR as components. Src thereafter phosphorylates AR at Tyr-534 residue to promote the nuclear translocation of AR, resulting in AR-driven transcription. Thus, this pathway acts in concert with classical mode of transcriptional activation of AR (52).

## Role of SHRs in Cell Cycle Regulation

The unanimous role of SHRs as transcription factors is widely worked upon in various research findings (4, 8, 53). Additionally, its non-genomic role is also established through a number of elegant works (20, 21). Among these numerous findings, we focused on those directly relevant to the regulation of cell cycle.

### G1 Phase

This phase regulates the rapid growth of eukaryotic cells, and is governed by a series of restrictive events. Cyclin D1 is an ER-responsive gene and this activation is of utmost importance in cellular progression through G1 phase. Ligand-bound-ER binds to an ERE site on the *CCND1* (Cyclin D1 coding) promoter to induce its expression. Cyclin D1 associates with CDK4/6, which in turn hyperphosphorylates and inactivates the retinoblastoma (Rb) tumor suppressor protein, thereby promoting G1/S transition of the cells (54, 55). Another ER-responsive gene is *c-MYC* that is involved in G1/S transition, acting in parallel to pRb/E2F pathway (56). ER, along with AP-1 transcription factors, can activate an upstream enhancer element of *c-MYC* promoter (57). Upon nuclear translocation, Estrogen-activated dimeric ER weakly seats on a half ERE present in the enhancer. This complex is stabilized by the interaction of ER and AP-1 factors (JunD and FosB), which further bind to the AP1 element. The resulting complex transactivates *MYC* gene expression (57). Similarly, ER-α interacts with p21-bound-Cyclin E/Cdk2 to cause the dissociation of p21 and the active Cyclin E/Cdk2 phosphorylates Rb, allowing the progression of cells from the G1 to S phase (58). Moreover, two reports highlighted an ERE element independent (non-genomic) mode of *CCND1* trans-regulation by ER. One such finding documented that the ER-mediated activation of CCND1 occurs through Sp1 transcription factor binding at GC-rich promoter sequences (59), where the other report informed the recruitment of cAMP response element (CRE) on *CCND1* by the combination of c-Jun and ER (60). Moreover, several groups documented a direct crosstalk between ER and Cyclin D1. This interaction was shown to lead the ER-driven transcription which is independent of Cyclin D1 association with Cdk4 and unrelated to the presence of hormone ligand (61–64).

Contrary to the role of ER in Cyclin D1 upregulation and G1 progression, the ligand-activated androgen receptor (AR) decreases the transcriptional activity of Cyclin D1. This reduction in Cyclin D1 levels is mediated by binding of AR in combination with an orphan nuclear receptor, DAX1, to an androgen response element (ARE) located on the *CCND1* proximal promoter (65). Interestingly, AR and ER-α actively compete to interact with a steroid receptor coactivator, A1B1. This coactivator molecule acts as the functional determinant of ER-α, facilitating its useful coupling with the *CCND1* promoter (66). The transcriptional activity of AR is markedly reduced at G1/S transition. This is due to downregulation of receptor levels and involvement of histone deacetylase that brings about chromatin remodeling to repress transcription (67). Hence, it could be assumed that in absence of AR activity, ER continues to trans-activate Cyclin D1 and thus aids in the cellular progression through G1 phase. On the separate note, the activity of the progesterone receptor is markedly reduced in G1 part, owing to the influence of histone deacetylases (68).

### S Phase

Cyclin A, in association with Cdk2 or Cdk1, regulates cellular progression through S phase *via* its phosphorylation activities. Among the substrates of this S-phase kinase, ER-α is also reported. ER-α is positively regulated by Cyclin A/Cdk2-mediated phosphorylation at its AF-1 domain (55, 69) and this indicates for cell cycle specific activity of ER-α. Overexpression of Cyclin A2 also increases AR activity (70). Reportedly, PR activity was also highest in S part (68). Research findings showed a significant increase of nuclear PR as a result of hormone treatment, exclusively at S phase (68). The ligand-independent phosphorylation of PR-B at S81 residue is catalyzed by Casein Kinase 2 (CK2), resulting in pronounced PR function during S phase (71). The transcriptional activity of PR is regulated by Cyclin A/Cdk2 (72). However, the ablation of phosphorylation sites found in the Ser/Thr-Pro motif of PR didn’t significantly lower the Cyclin A/Cdk2-mediated kinase activity, suggesting an indirect mode of phosphorylation of PR. Cyclin A/Cdk2 can act as PR coactivator and the interaction of Cyclin A and PR was reported. The binding of Cyclin A/Cdk2 to the promoters of PR-responsive genes facilitates phosphorylation of associated proteins (72). An *in vitro* study reported the reduced binding of SRC-1 to PR upon phosphatase treatment (72); however, the interaction was restored by rephosphorylation with Cyclin A/Cdk2. The LXXLL motifs in SRC-1 mediate its interaction with PR (73). Therefore, PR-dependent recruitment of Cyclin A/Cdk2 is able to elicit a rise in kinase concentrations, required for phosphorylating SRC-1 on those motifs to trigger its PR-affinity (70, 72).

### G2 Phase

ER-α can modulate G2-M transition by repressing a cell cycle inhibitor, Reprimo (RPRM). ER-α, histone deacetylase 7 (HDAC7), and FoxA1 together form a complex to inhibit RPRM, to drive the cell cycle progression (55). In G2 phase, the transcriptional activity of PR is significantly reduced, and site-specific phosphorylation of PR at Ser162 and Ser294 is abolished (68). HSPB8, a heat-shock protein at the G2/M phase requires cyclin D1 for its expression (74). The expression and transcriptional activity of AR largely depends on the CDK1-mediated phosphorylation, which stabilizes AR (75); however, androgen-stimulated transactivation is absent in G2/M (76). The transcriptional activities of steroid receptors can be regulated by the involvement of G2/M kinases. Such a kinase, named PLK1 (polo-like kinase 1) communicates with ER-α and modulates the transcription of ER-α target genes in breast cancer cells (77). Similarly, Aurora A, a serine/threonine kinase, is involved in phosphorylation of ER-α at S167 and S305, a modification involved in transcriptional activity of ER-α (78).

### M Phase

**Figure 3:**
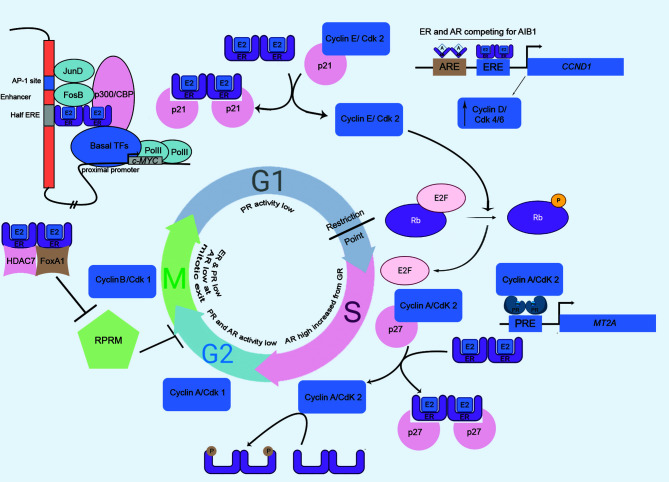
The cell cycle, driven by the master regulators: steroid hormone receptors and kinases. SHRs involve in various cell cycle phase-specific functions, engaging the retinoblastoma protein (Rb), cyclins, and, cyclin-dependent kinases (cdk). MT2A, Metallothionein IIA, a PR target gene; A, Androgen; Pro, Progesterone; ERE, Estrogen Response Element; PRE, Progesterone Response Element; ARE, Androgen Response Element.

The accessibility of chromatin is largely reduced at M-phase. As the genome remains transcriptionally suppressed, no such role of SHR in M-phase is documented (**Figure 3**).

## SHR-Driven Cross-Link Between Cell Cycle and Breast Cancer

Breast cancer is the commonest malignancy in women across the world and predominantly related to cancer-related deaths in women (79). Breast cancer can be subdivided as either luminal (ER and/or PR+ve), human epidermal growth factor receptor 2 (HER2)-enriched or triple negative (TNBC) subtype (ER, PR, and HER2 −ve) (80). Over the previous decades, the insights into the molecular heterogeneity of this dreaded disease have been extensively divulged and the integral role of cell cycle machinery in oncogenesis as well as hormone therapy resistance has picked up expanding consideration. In the following section, this review will elaborate the current knowledge on the role of breast cancer associated SHR members (specially emphasizing ER, PR, and AR) in oncogenesis of breast through deregulation of cell cycle.

### Cell Cycle Anomalies in Breast Cancer Development and Progression

The safeguards and checkpoints of the cell cycle are often overridden in cancer cells. A number of molecular alterations in several checkpoints of the cell cycle are noticeable in the different subtypes of breast cancer (81).

Luminal tumors (ER and/or PR+ve) are generally characterized by an enhanced expression of Cyclin D1, *via* estrogen receptor activation that binds directly to the *CCND1* promoter, enhancing its expression (82, 83). This cellular phenomenon is surely a prerequisite step for cell cycle progression, while ER upregulation might bring cellular hyper-proliferation upon influencing Cyclin D1 overexpression. This might be the case in breast cancers where *CCND1* gene is overexpressed in at least 50% of incidences (84). Indeed, E2-stimulated Cyclin D1 expression in ER+ve breast cancer cells largely relies on an enhancer element downstream of *CCND1* coding sequence, corresponding to the predominant ER-α recruiting site. FoxA1 mediates chromatin remodeling to activate this enhancer, and drives ER-α-mediated *CCND1* expression and concomitant cell proliferation, upon recruiting additional transcription factors, such as Sp1. Simultaneously, a tight regulation of Cyclin D1-mediated cell proliferation is also observed at cellular level. NFIC, a tumor suppressor, directly represses. the transcriptional activity of Cyclin D1, upon an E2 stimulus (85). It could be well assumed that the loss of tumor suppressor, as frequent in breast cancer cells, might contribute to the ER-CyclinD1 crosstalk and bring forward cellular malignancy. Furthermore, in ER+ve breast cancer models, dihydrotestosterone (DHT)-mediated activation of AR has been shown to inhibit ER-α signaling and cell cycle progression through a reduction in *CCND1* transcription (66). There involves a competition in transcriptional activity of AR and ER-α that is more precisely dependent on the bio-availability of AIB1, a steroid receptor coactivator. It was reported that the rise in AR intracellular concentration dictated a marked decrease in E2-induced AIB1 recruitment at the AP1 site of *CCND1* promoter (66), resulting in reduced ER-driven transcription of *CCND1* and concomitant inhibition of estrogen-induced cell proliferation (86). On the other hand, in AR+ TNBC, DHT has been shown to increase levels of Cyclin D1, while decreasing p73 and p21 expression (7). p21 is a Cyclin-dependent kinase that involves in tumor suppression by cell cycle arrest and its downregulation was reported to cause breast cancer (87). AR blocks the promoter regions of p73 and p21 for their co-activators, leading to the downregulation of these two tumor suppressors. These studies experimentally validated the influence of AR on TNBC cell proliferation, exploiting the above mechanism (7). Additionally, the luminal androgen receptor (LAR) subgroup, a subtype of TNBC, might be sensitive to inhibition of CDK4/6 pathway due to the association of AR expression and RB1 expression (81, 88). The Cyclin E overexpression in TNBC exhibits a poor prognostic significance and is associated with the absence of ER and PR (89). This finding is concordant with a number of studies (90, 91), one of which depicted the correlation of highly expressed Cyclin E in steroid receptor negative tumors with the failure of endocrine therapy (91).

### Targeting the Cell Cycle in Breast Cancer

**Figure 4:**
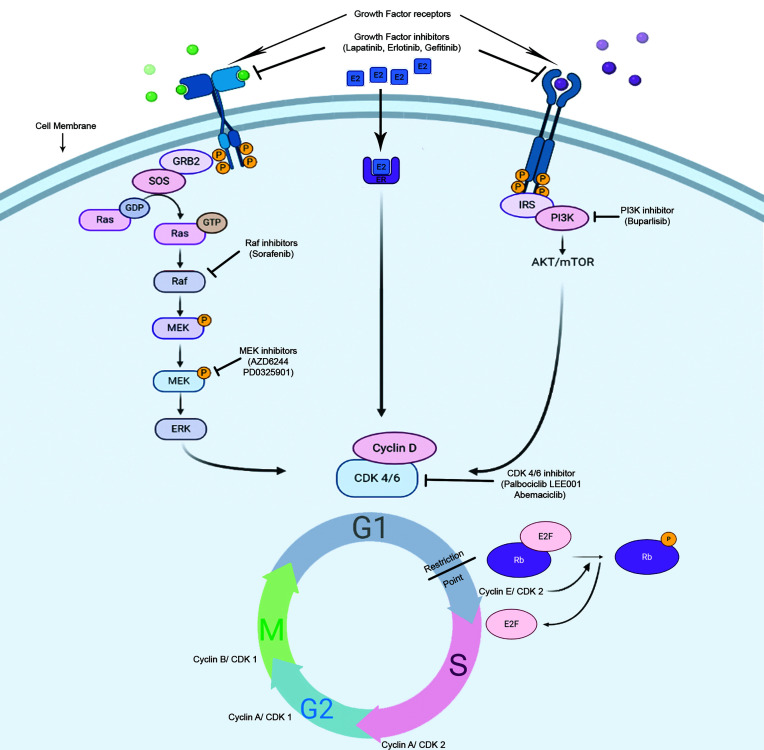
Potential mode of action of cell cycle inhibitors in hormone-responsive breast tumors. There are numerous anti-cancer strategies that specifically target cell proliferation in breast cancer. The dependence of the HR+ve cells on Rb/E2F/CDK4/6 axis for progression through restriction point makes them vulnerable for CDK4/6 inhibitors.

*CDK4/6 inhibitors in SHR-positive breast cancer:* Due to the central role of Cyclin D/CDK4/6 complex in the control of the breast cancer proliferation and in the estrogen receptor signaling pathway, several CDK4/6 inhibitors have been investigated as breast cancer therapeutics in the last decades, in particular in luminal breast cancer (**Figure 4**).

Selective ATP-competitive inhibitors of CDK4/6, including palbociclib, ribociclib, and abemaciclib have recently been evaluated in clinical trials in combination with standard endocrine therapies in metastatic breast cancer, and have demonstrated significant improvements in tumor response rate and progression-free survival compared to endocrine therapy alone, which has led to their FDA approvals in combination with aromatase inhibitors or fulvestrant (92, 93). The steroid hormone receptor-positive breast cancer cell lines show the most sensitivity towards palbociclib (94). In estrogen-responsive cells, the effects of estrogen on the cell cycle progression depend highly on Cyclin D1 expression (95). In ER+ve breast tumors, there is an increased expression of Cyclin D1 or CCND1 gene amplification, as well as, p16 or Rb losses (81, 96). These molecular signatures predicted the sensitivity of ER+ve breast cancer towards palbociclib (94). This drug effectively arrests the cells at G1-S boundary and inhibits cell proliferation, in Rb-positive cells of different tumor types, with concomitant Rb dephosphorylation at specific serine residues (Ser780/Ser795) (97).

*CDK4/6 inhibitors in triple negative breast cancer:* A major challenge in developing targeted therapies in TNBC patients dwells on the variable level of response to treatments which reflects underlying heterogeneity within this subtype. Advances in the knowledge of gene expression profiling of TNBC have led to complementary classification systems that may be associated with response to therapy agents (98). For example, new insights in TNBC classification profiling isolated a subset of TNBC that is enriched for Androgen Receptor (AR) expression. AR positive TNBC are particularly sensitive to CDK 4/6 inhibition. The molecular mechanism underpinning this observation depends on cells bearing two different subpopulations of CDK2, CDK2-high and CDK2-low, dictating a biphasic mitotic exit of cells into proliferative and quiescent state, respectively (99, 100). Palbociclib-sensitive LAR (Luminal androgen receptor positive; a subtype of TNBC) cancer cells typically exit into a quiescent CDK2^low^ state post-mitosis, from which CDK4/6 is required to phosphorylate RB1 and pass the restriction point (101–103). The requirement of CDK4/6 sensitizes these LAR cells towards CDK4/6 inhibitors. This led several ongoing trials to clinically evaluate the role of the antiandrogen drug bicalutamide in combination with CDK inhibitors in AR-positive metastatic breast cancer (104).

Complex relations exist between Rb protein, TNBC and AR expression. In fact, although Rb is commonly lost in TNBC, studies showed an association between Rb and AR expression in those tumors and sensitivity to palbociclib was seen in luminal androgen receptor (LAR) cell lines (105–107). A prior study reported that AR antagonist enzalutamide enhanced the palbociclib-induced G1 arrest in AR-positive/RB-proficient (MDA-MB-453) cells rather than AR-positive/RB-negative (BT-549), AR-negative/RB-proficient (MDA-MB-231), and AR-negative/RB-negative (MDA-MB-468) cells (108). It was previously documented that the interaction of RB with AR is androgen-independent and the protein functions as an AR-coactivator (109). Moreover, DNA replication is indirectly stimulated by AR through hyperphosphorylated RB (110). Palbociclib decreases the phosphorylation of RB, and enzalutamide might decrease the RB coactivator recruitment leading to RB-mediated cell cycle arrest.

## Discussion

In recent years, increasing evidence implicating SHR in cross-talk with cell cycle anomalies in breast cancer pathogenesis has elicited immense interest in understanding the combinatorial treatment options exploiting breast cancer specific SHR members with cell cycle inhibitors. SHR-induced deregulation in cell-cycle control and the subsequent breast cancer progression unraveled a potential unexplored regimen in breast cancer treatment by introducing several clinical trials with cell-cycle inhibitors in clinical practice (111). In this era of precision medicine, different CDK inhibitors have emerged as novel therapeutics due to their selective abrogation of only the cancer cells. Although targeted monotherapies have resulted in favored outcome in the clinic, the emergence of acquired therapy-resistance is almost unavoidable. To overcome these circumstances, the selective drug combinations (combinatorial drug therapy) will be the only option to control the disease in a more efficacious manner. Until recently, several CDK4/6 inhibitors have been clinically developed and further understandings will be required to administer optimal combination of these agents with the other highly selective SHR-related therapeutics towards a better management of breast cancer.

## Author Contributions

SS and SN conceptualized and wrote the manuscript. SD wrote the manuscript. All authors contributed to the article and approved the submitted version.

## Funding

SS is supported by Department of Science & Technology—INSPIRE Senior Research Fellowship (IF170820) and SN is supported by Early Career Award, Science & Engineering Research Board (SERB)—Department of Science and Technology (DST), Government of India (File No. ECR/2015/000206), and Grant-in-Aid, Department of Science & Technology and Biotechnology (DSTBT), Government of West Bengal (ST/P/S&T/9G-21/2016), and Extra Mural Grant, Science & Engineering Research Board (SERB) - Department of Science and Technology (DST), Government of India (File No. EMR/2015/001835).

## Conflict of Interest

The authors declare that the research was conducted in the absence of any commercial or financial relationships that could be construed as a potential conflict of interest.

## References

[1] Barnum KJ O’Connell MJ. Cell cycle regulation by checkpoints. Methods Mol Biol (2014) 1170:29–40. 10.1007/978-1-4939-0888-2_2 24906307 PMC4990352

[2] Visconti R Della Monica R Grieco D. Cell cycle checkpoint in cancer: a therapeutically targetable double-edged sword. J Exp Clin Cancer Res (2016) 35:153. 10.1186/s13046-016-0433-9 27670139 PMC5037895

[3] Wenzel ES Singh ATK. Cell-cycle Checkpoints and Aneuploidy on the Path to Cancer. In Vivo (2018) 32:1–5. 10.21873/invivo.11197 29275292 PMC5892633

[4] Beato M Klug J. Steroid hormone receptors: an update. Hum Reprod Update (2000) 6:225–36. 10.1093/humupd/6.3.225 10874567

[5] Truong TH Lange CA. Deciphering Steroid Receptor Crosstalk in Hormone-Driven Cancers. Endocrinology (2018) 159:3897–907. 10.1210/en.2018-00831 PMC6236424 30307542

[6] Tanos T Rojo L Echeverria P Brisken C. ER and PR signaling nodes during mammary gland development. Breast Cancer Res (2012) 14:210. 10.1186/bcr3166 22809143 PMC3680919

[7] Zhu A Li Y Song W Xu Y Yang F Zhang W. Antiproliferative Effect of Androgen Receptor Inhibition in Mesenchymal Stem-Like Triple-Negative Breast Cancer. Cell Physiol Biochem (2016) 38:1003–14. 10.1159/000443052 26938985

[8] Klinge CM. Steroid hormone receptors and signal transduction processes. In: Principles of Endocrinology Hormone Action. Springer International Publishing (2018). p. 187.

[9] Warnmark A Treuter E Wright AP Gustafsson JA. Activation functions 1 and 2 of nuclear receptors: molecular strategies for transcriptional activation. Mol Endocrinol (2003) 17:1901–9. 10.1210/me.2002-0384 12893880

[10] Lavery DN McEwan IJ. Structure and function of steroid receptor AF1 transactivation domains: induction of active conformations. Biochem J (2005) 391:449–64. 10.1042/BJ20050872 PMC1276946 16238547

[11] Weikum ER Liu X Ortlund EA. The nuclear receptor superfamily: A structural perspective. Protein Sci (2018) 27:1876–92. 10.1002/pro.3496 PMC6201731 30109749

[12] Rastinejad F Huang P Chandra V Khorasanizadeh S. Understanding nuclear receptor form and function using structural biology. J Mol Endocrinol (2013) 51:T1–T21. 10.1530/JME-13-0173 24103914 PMC3871882

[13] Weigel NL Moore NL. Kinases and protein phosphorylation as regulators of steroid hormone action. Nucl Recept Signal (2007) 5:e005. 10.1621/nrs.05005 17525795 PMC1876600

[14] Obeid JP Zafar N El Hokayem J. Steroid Hormone Receptor Coregulators in Endocrine Cancers. IUBMB Life (2016) 68:504–15. 10.1002/iub.1517 27240871

[15] Green S Walter P Kumar V Krust A Bornert JM Argos P. Human oestrogen receptor cDNA: sequence, expression and homology to v-erb-A. Nature (1986) 320:134–9. 10.1038/320134a0 3754034

[16] Mosselman S Polman J Dijkema R. ER beta: identification and characterization of a novel human estrogen receptor. FEBS Lett (1996) 392:49–53. 10.1016/0014-5793(96)00782-X 8769313

[17] Fuentes N Silveyra P. Estrogen receptor signaling mechanisms. Adv Protein Chem Struct Biol (2019) 116:135–70. 10.1016/bs.apcsb.2019.01.001 PMC6533072 31036290

[18] Scarpin KM Graham JD Mote PA Clarke CL. Progesterone action in human tissues: regulation by progesterone receptor (PR) isoform expression, nuclear positioning and coregulator expression. Nucl Recept Signal (2009) 7:e009. 10.1621/nrs.07009 20087430 PMC2807635

[19] Davey RA Grossmann M. Androgen Receptor Structure, Function and Biology: From Bench to Bedside. Clin Biochem Rev (2016) 37:3–15. 27057074 PMC4810760

[20] Levin ER Hammes SR. Nuclear receptors outside the nucleus: extranuclear signalling by steroid receptors. Nat Rev Mol Cell Biol (2016) 17:783–97. 10.1038/nrm.2016.122 PMC5649368 27729652

[21] Levin ER. Extranuclear steroid receptors are essential for steroid hormone actions. Annu Rev Med (2015) 66:271–80. 10.1146/annurev-med-050913-021703 25587652

[22] Pedram A Razandi M Deschenes RJ Levin ER. DHHC-7 and -21 are palmitoylacyltransferases for sex steroid receptors. Mol Biol Cell (2012) 23:188–99. 10.1091/mbc.e11-07-0638 PMC3248897 22031296

[23] Pedram A Razandi M Sainson RC Kim JK Hughes CC Levin ER. A conserved mechanism for steroid receptor translocation to the plasma membrane. J Biol Chem (2007) 282:22278–88. 10.1074/jbc.M611877200 17535799

[24] Pedram A Razandi M Levin ER. Nature of functional estrogen receptors at the plasma membrane. Mol Endocrinol (2006) 20:1996–2009. 10.1210/me.2005-0525 16645038

[25] Losel R Wehling M. Nongenomic actions of steroid hormones. Nat Rev Mol Cell Biol (2003) 4:46–56. 10.1038/nrm1009 12511868

[26] Cato AC Nestl A Mink S. Rapid actions of steroid receptors in cellular signaling pathways. Sci STKE (2002) 2002:re9. 10.1126/stke.2002.138.re9 12084906

[27] Chambliss KL Yuhanna IS Mineo C Liu P German Z Sherman TS. Estrogen receptor alpha and endothelial nitric oxide synthase are organized into a functional signaling module in caveolae. Circ Res (2000) 87:E44–52. 10.1161/01.RES.87.11.e44 11090554

[28] Hammes SR Levin ER. Extranuclear steroid receptors: nature and actions. Endocr Rev (2007) 28:726–41. 10.1210/er.2007-0022 17916740

[29] Zheng FF Wu RC Smith CL O’Malley BW. Rapid estrogen-induced phosphorylation of the SRC-3 coactivator occurs in an extranuclear complex containing estrogen receptor. Mol Cell Biol (2005) 25:8273–84. 10.1128/MCB.25.18.8273-8284.2005 PMC1234335 16135815

[30] Su X Xu X Li G Lin B Cao J Teng L. ER-alpha36: a novel biomarker and potential therapeutic target in breast cancer. Onco Targets Ther (2014) 7:1525–33. 10.2147/OTT.S65345 PMC4155893 25210466

[31] Gaudet HM Cheng SB Christensen EM Filardo EJ. The G-protein coupled estrogen receptor, GPER: The inside and inside-out story. Mol Cell Endocrinol (2015) 418 Pt 3:207–19. 10.1016/j.mce.2015.07.016 26190834

[32] Prossnitz ER Barton M. Estrogen biology: new insights into GPER function and clinical opportunities. Mol Cell Endocrinol (2014) 389:71–83. 10.1016/j.mce.2014.02.002 24530924 PMC4040308

[33] Garg D Ng SSM Baig KM Driggers P Segars J. Progesterone-Mediated Non-Classical Signaling. Trends Endocrinol Metab (2017) 28:656–68. 10.1016/j.tem.2017.05.006 28651856

[34] Filardo EJ Thomas P. Minireview: G protein-coupled estrogen receptor-1, GPER-1: its mechanism of action and role in female reproductive cancer, renal and vascular physiology. Endocrinology (2012) 153:2953–62. 10.1210/en.2012-1061 PMC3380306 22495674

[35] Girgert R Emons G Grundker C. 17beta-estradiol-induced growth of triple-negative breast cancer cells is prevented by the reduction of GPER expression after treatment with gefitinib. Oncol Rep (2017) 37:1212–8. 10.3892/or.2016.5306 27959426

[36] Boonyaratanakornkit V Hamilton N Marquez-Garban DC Pateetin P McGowan EM Pietras RJ. Extranuclear signaling by sex steroid receptors and clinical implications in breast cancer. Mol Cell Endocrinol (2018) 466:51–72. 10.1016/j.mce.2017.11.010 29146555 PMC5878997

[37] Sjostrom M Hartman L Grabau D Fornander T Malmstrom P Nordenskjold B. Lack of G protein-coupled estrogen receptor (GPER) in the plasma membrane is associated with excellent long-term prognosis in breast cancer. Breast Cancer Res Treat (2014) 145:61–71. 10.1007/s10549-014-2936-4 24715381

[38] Thomas P Pang Y Dong J Groenen P Kelder J de Vlieg J. Steroid and G protein binding characteristics of the seatrout and human progestin membrane receptor alpha subtypes and their evolutionary origins. Endocrinology (2007) 148:705–18. 10.1210/en.2006-0974 17082257

[39] Boonyaratanakornkit V Scott MP Ribon V Sherman L Anderson SM Maller JL. Progesterone receptor contains a proline-rich motif that directly interacts with SH3 domains and activates c-Src family tyrosine kinases. Mol Cell (2001) 8:269–80. 10.1016/S1097-2765(01)00304-5 11545730

[40] Karteris E Zervou S Pang Y Dong J Hillhouse EW Randeva HS. Progesterone signaling in human myometrium through two novel membrane G protein-coupled receptors: potential role in functional progesterone withdrawal at term. Mol Endocrinol (2006) 20:1519–34. 10.1210/me.2005-0243 16484338

[41] Pace MC Thomas P. Steroid-induced oocyte maturation in Atlantic croaker (Micropogonias undulatus) is dependent on activation of the phosphatidylinositol 3-kinase/Akt signal transduction pathway. Biol Reprod (2005) 73:988–96. 10.1095/biolreprod.105.041400 16014813

[42] Tubbs CW. Mechanisms of progestin-stimulated sperm hypermotility in two teleosts: the Atlantic croaker (Micropogonias undulatus) and the southern flounder (Platylicthys lethigstomata). (2007).

[43] Ashley RL Clay CM Farmerie TA Niswender GD Nett TM. Cloning and characterization of an ovine intracellular seven transmembrane receptor for progesterone that mediates calcium mobilization. Endocrinology (2006) 147:4151–9. 10.1210/en.2006-0002 16794007

[44] Falkenstein E Heck M Gerdes D Grube D Christ M Weigel M. Specific progesterone binding to a membrane protein and related nongenomic effects on Ca2+-fluxes in sperm. Endocrinology (1999) 140:5999–6002. 10.1210/endo.140.12.7304 10579369

[45] Cahill MA. Progesterone receptor membrane component 1: an integrative review. J Steroid Biochem Mol Biol (2007) 105:16–36. 10.1016/j.jsbmb.2007.02.002 17583495

[46] Cinar B Mukhopadhyay NK Meng G Freeman MR. Phosphoinositide 3-kinase-independent non-genomic signals transit from the androgen receptor to Akt1 in membrane raft microdomains. J Biol Chem (2007) 282:29584–93. 10.1074/jbc.M703310200 17635910

[47] Garza-Contreras J Duong P Snyder BD Schreihofer DA Cunningham RL. Presence of Androgen Receptor Variant in Neuronal Lipid Rafts. eNeuro (2017) 4. 10.1523/ENEURO.0109-17.2017 PMC5575139 28856243

[48] Pedram A Razandi M O’Mahony F Harvey H Harvey BJ Levin ER. Estrogen reduces lipid content in the liver exclusively from membrane receptor signaling. Sci Signal (2013) 6:ra36. 10.1126/scisignal.2004013 23695162

[49] Pedram A Razandi M Lewis M Hammes S Levin ER. Membrane-localized estrogen receptor α is required for normal organ development and function. Dev Cell (2014) 29:482–90. 10.1016/j.devcel.2014.04.016 PMC4062189 24871949

[50] Giovannelli P Di Donato M Auricchio F Castoria G Migliaccio A. Androgens Induce Invasiveness of Triple Negative Breast Cancer Cells Through AR/Src/PI3-K Complex Assembly. Sci Rep (2019) 9:4490. 10.1038/s41598-019-41016-4 30872694 PMC6418124

[51] Giovannelli P Di Donato M Galasso G Di Zazzo E Bilancio A Migliaccio A. The Androgen Receptor in Breast Cancer. Front Endocrinol (Lausanne) (2018) 9:492. 10.3389/fendo.2018.00492 30210453 PMC6122126

[52] Yang X Guo Z Sun F Li W Alfano A Shimelis H. Novel membrane-associated androgen receptor splice variant potentiates proliferative and survival responses in prostate cancer cells. J Biol Chem (2011) 286:36152–60. 10.1074/jbc.M111.265124 PMC3195613 21878636

[53] Kininis M Kraus WL. A global view of transcriptional regulation by nuclear receptors: gene expression, factor localization, and DNA sequence analysis. Nucl Recept Signal (2008) 6:e005. 10.1621/nrs.06005 18301785 PMC2254333

[54] Lukas J Bartkova J Bartek J. Convergence of mitogenic signalling cascades from diverse classes of receptors at the cyclin D-cyclin-dependent kinase-pRb-controlled G1 checkpoint. Mol Cell Biol (1996) 16:6917–25. 10.1128/MCB.16.12.6917 PMC231695 8943347

[55] Moghadam SJ Hanks AM Keyomarsi K. Breaking the cycle: An insight into the role of ERalpha in eukaryotic cell cycles. J Carcinog (2011) 10:25. 10.4103/1477-3163.90440 22190867 PMC3243079

[56] Santoni-Rugiu E Falck J Mailand N Bartek J Lukas J. Involvement of Myc activity in a G(1)/S-promoting mechanism parallel to the pRb/E2F pathway. Mol Cell Biol (2000) 20:3497–509. 10.1128/MCB.20.10.3497-3509.2000 PMC85642 10779339

[57] Wang C Mayer JA Mazumdar A Fertuck K Kim H Brown M. Estrogen induces c-myc gene expression *via an* upstream enhancer activated by the estrogen receptor and the AP-1 transcription factor. Mol Endocrinol (2011) 25:1527–38. 10.1210/me.2011-1037 PMC3165912 21835891

[58] Prall OW Sarcevic B Musgrove EA Watts CK Sutherland RL. Estrogen-induced activation of Cdk4 and Cdk2 during G1-S phase progression is accompanied by increased cyclin D1 expression and decreased cyclin-dependent kinase inhibitor association with cyclin E-Cdk2. J Biol Chem (1997) 272:10882–94. 10.1074/jbc.272.16.10882 9099745

[59] Castro-Rivera E Samudio I Safe S. Estrogen regulation of cyclin D1 gene expression in ZR-75 breast cancer cells involves multiple enhancer elements. J Biol Chem (2001) 276:30853–61. 10.1074/jbc.M103339200 11410592

[60] Sabbah M Courilleau D Mester J Redeuilh G. Estrogen induction of the cyclin D1 promoter: involvement of a cAMP response-like element. Proc Natl Acad Sci U S A (1999) 96:11217–22. 10.1073/pnas.96.20.11217 PMC18014 10500157

[61] McMahon C Suthiphongchai T DiRenzo J Ewen ME. P/CAF associates with cyclin D1 and potentiates its activation of the estrogen receptor. Proc Natl Acad Sci U S A (1999) 96:5382–7. 10.1073/pnas.96.10.5382 PMC21868 10318892

[62] Neuman E Ladha MH Lin N Upton TM Miller SJ DiRenzo J. Cyclin D1 stimulation of estrogen receptor transcriptional activity independent of cdk4. Mol Cell Biol (1997) 17:5338–47. 10.1128/MCB.17.9.5338 PMC232384 9271411

[63] Zwijsen RM Buckle RS Hijmans EM Loomans CJ Bernards R. Ligand-independent recruitment of steroid receptor coactivators to estrogen receptor by cyclin D1. Genes Dev (1998) 12:3488–98. 10.1101/gad.12.22.3488 PMC317237 9832502

[64] Zwijsen RM Wientjens E Klompmaker R van der Sman J Bernards R Michalides RJ. CDK-independent activation of estrogen receptor by cyclin D1. Cell (1997) 88:405–15. 10.1016/S0092-8674(00)81879-6 9039267

[65] Lanzino M Sisci D Morelli C Garofalo C Catalano S Casaburi I. Inhibition of cyclin D1 expression by androgen receptor in breast cancer cells–identification of a novel androgen response element. Nucleic Acids Res (2010) 38:5351–65. 10.1093/nar/gkq278 PMC2938215 20421209

[66] De Amicis F Chiodo C Morelli C Casaburi I Marsico S Bruno R. AIB1 sequestration by androgen receptor inhibits estrogen-dependent cyclin D1 expression in breast cancer cells. BMC Cancer (2019) 19:1038. 10.1186/s12885-019-6262-4 31684907 PMC6829973

[67] Martinez ED Danielsen M. Loss of androgen receptor transcriptional activity at the G(1)/S transition. J Biol Chem (2002) 277:29719–29. 10.1074/jbc.M112134200 12055183

[68] Narayanan R Edwards DP Weigel NL. Human progesterone receptor displays cell cycle-dependent changes in transcriptional activity. Mol Cell Biol (2005) 25:2885–98. 10.1128/MCB.25.8.2885-2898.2005 PMC1069605 15798179

[69] Rogatsky I Trowbridge JM Garabedian MJ. Potentiation of human estrogen receptor alpha transcriptional activation through phosphorylation of serines 104 and 106 by the cyclin A-CDK2 complex. J Biol Chem (1999) 274:22296–302. 10.1074/jbc.274.32.22296 10428798

[70] Weigel NL Moore NL. Cyclins, cyclin dependent kinases, and regulation of steroid receptor action. Mol Cell Endocrinol (2007) 265-266:157–61. 10.1016/j.mce.2006.12.013 PMC1940111 17207919

[71] Hagan CR Regan TM Dressing GE Lange CA. ck2-dependent phosphorylation of progesterone receptors (PR) on Ser81 regulates PR-B isoform-specific target gene expression in breast cancer cells. Mol Cell Biol (2011) 31:2439–52. 10.1128/MCB.01246-10 PMC3133426 21518957

[72] Narayanan R Adigun AA Edwards DP Weigel NL. Cyclin-dependent kinase activity is required for progesterone receptor function: novel role for cyclin A/Cdk2 as a progesterone receptor coactivator. Mol Cell Biol (2005) 25:264–77. 10.1128/MCB.25.1.264-277.2005 PMC538783 15601848

[73] Heery DM Hoare S Hussain S Parker MG Sheppard H. Core LXXLL motif sequences in CREB-binding protein, SRC1, and RIP140 define affinity and selectivity for steroid and retinoid receptors. J Biol Chem (2001) 276:6695–702. 10.1074/jbc.M009404200 11078741

[74] Dressing GE Knutson TP Schiewer MJ Daniel AR Hagan CR Diep CH. Progesterone receptor-cyclin D1 complexes induce cell cycle-dependent transcriptional programs in breast cancer cells. Mol Endocrinol (2014) 28:442–57. 10.1210/me.2013-1196 PMC3968407 24606123

[75] Chen S Xu Y Yuan X Bubley GJ Balk SP. Androgen receptor phosphorylation and stabilization in prostate cancer by cyclin-dependent kinase 1. Proc Natl Acad Sci U S A (2006) 103:15969–74. 10.1073/pnas.0604193103 PMC1635111 17043241

[76] Koryakina Y Knudsen KE Gioeli D. Cell-cycle-dependent regulation of androgen receptor function. Endocr Relat Cancer (2015) 22:249–64. 10.1530/ERC-14-0549 PMC4382102 25691442

[77] Wierer M Verde G Pisano P Molina H Font-Mateu J Di Croce L. PLK1 signaling in breast cancer cells cooperates with estrogen receptor-dependent gene transcription. Cell Rep (2013) 3:2021–32. 10.1016/j.celrep.2013.05.024 23770244

[78] Opyrchal M Salisbury JL Zhang S McCubrey J Hawse J Goetz MP. Aurora-A mitotic kinase induces endocrine resistance through down-regulation of ERalpha expression in initially ERalpha+ breast cancer cells. PloS One (2014) 9:e96995. 10.1371/journal.pone.0096995 24816249 PMC4016211

[79] Ferlay J Shin HR Bray F Forman D Mathers C Parkin DM. Estimates of worldwide burden of cancer in 2008: GLOBOCAN 2008. Int J Cancer (2010) 127:2893–917. 10.1002/ijc.25516 21351269

[80] Dai X Li T Bai Z Yang Y Liu X Zhan J. Breast cancer intrinsic subtype classification, clinical use and future trends. Am J Cancer Res (2015) 5:2929–43. PMC4656721 26693050

[81] N. Cancer Genome Atlas. Comprehensive molecular portraits of human breast tumours. Nature (2012) 490:61–70. 10.1038/nature11412 23000897 PMC3465532

[82] VanArsdale T Boshoff C Arndt KT Abraham RT. Molecular Pathways: Targeting the Cyclin D-CDK4/6 Axis for Cancer Treatment. Clin Cancer Res (2015) 21:2905–10. 10.1158/1078-0432.CCR-14-0816 25941111

[83] Peurala E Koivunen P Haapasaari KM Bloigu R Jukkola-Vuorinen A. The prognostic significance and value of cyclin D1, CDK4 and p16 in human breast cancer. Breast Cancer Res (2013) 15:R5. 10.1186/bcr3376 23336272 PMC3672746

[84] Arnold A Papanikolaou A. Cyclin D1 in breast cancer pathogenesis. J Clin Oncol (2005) 23:4215–24. 10.1200/JCO.2005.05.064 15961768

[85] Eeckhoute J Carroll JS Geistlinger TR Torres-Arzayus MI Brown M. A cell-type-specific transcriptional network required for estrogen regulation of cyclin D1 and cell cycle progression in breast cancer. Genes Dev (2006) 20:2513–26. 10.1101/gad.1446006 PMC1578675 16980581

[86] Planas-Silva MD Shang Y Donaher JL Brown M Weinberg RA. AIB1 enhances estrogen-dependent induction of cyclin D1 expression. Cancer Res (2001) 61:3858–62. 11358796

[87] Bachman KE Blair BG Brenner K Bardelli A Arena S Zhou S. p21(WAF1/CIP1) mediates the growth response to TGF-beta in human epithelial cells. Cancer Biol Ther (2004) 3:221–5. 10.4161/cbt.3.2.666 14726675

[88] Spring LM Wander SA Andre F Moy B Turner NC Bardia A. Cyclin-dependent kinase 4 and 6 inhibitors for hormone receptor-positive breast cancer: past, present, and future. Lancet (2020) 395:817–27. 10.1016/S0140-6736(20)30165-3 32145796

[89] Jabbour-Leung NA Chen X Bui T Jiang Y Yang D Vijayaraghavan S. Sequential Combination Therapy of CDK Inhibition and Doxorubicin Is Synthetically Lethal in p53-Mutant Triple-Negative Breast Cancer. Mol Cancer Ther (2016) 15:593–607. 10.1158/1535-7163.MCT-15-0519 26826118 PMC4873336

[90] Donnellan R Kleinschmidt I Chetty R. Cyclin E immunoexpression in breast ductal carcinoma: pathologic correlations and prognostic implications. Hum Pathol (2001) 32:89–94. 10.1053/hupa.2001.21141 11172300

[91] Span PN Tjan-Heijnen VC Manders P Beex LV Sweep CG. Cyclin-E is a strong predictor of endocrine therapy failure in human breast cancer. Oncogene (2003) 22:4898–904. 10.1038/sj.onc.1206818 12894232

[92] Rocca A Schirone A Maltoni R Bravaccini S Cecconetto L Farolfi A. Progress with palbociclib in breast cancer: latest evidence and clinical considerations. Ther Adv Med Oncol (2017) 9:83–105. 10.1177/1758834016677961 28203301 PMC5298405

[93] Barroso-Sousa R Shapiro GI Tolaney SM. Clinical Development of the CDK4/6 Inhibitors Ribociclib and Abemaciclib in Breast Cancer. Breast Care (Basel) (2016) 11:167–73. 10.1159/000447284 PMC4960359 27493615

[94] Finn RS Dering J Conklin D Kalous O Cohen DJ Desai AJ. PD 0332991, a selective cyclin D kinase 4/6 inhibitor, preferentially inhibits proliferation of luminal estrogen receptor-positive human breast cancer cell lines *in vitro* . Breast Cancer Res (2009) 11:R77. 10.1186/bcr2419 19874578 PMC2790859

[95] Butt AJ McNeil CM Musgrove EA Sutherland RL. Downstream targets of growth factor and oestrogen signalling and endocrine resistance: the potential roles of c-Myc, cyclin D1 and cyclin E. Endocr Relat Cancer (2005) 12 Suppl 1:S47–59. 10.1677/erc.1.00993 16113099

[96] Witkiewicz AK Knudsen ES. Retinoblastoma tumor suppressor pathway in breast cancer: prognosis, precision medicine, and therapeutic interventions. Breast Cancer Res (2014) 16:207. 10.1186/bcr3652 25223380 PMC4076637

[97] Fry DW Harvey PJ Keller PR Elliott WL Meade M Trachet E. Specific inhibition of cyclin-dependent kinase 4/6 by PD 0332991 and associated antitumor activity in human tumor xenografts. Mol Cancer Ther (2004) 3:1427–38. 15542782

[98] Lehmann BD Bauer JA Chen X Sanders ME Chakravarthy AB Shyr Y. Identification of human triple-negative breast cancer subtypes and preclinical models for selection of targeted therapies. J Clin Invest (2011) 121:2750–67. 10.1172/JCI45014 PMC3127435 21633166

[99] Cappell SD Chung M Jaimovich A Spencer SL Meyer T. Irreversible APC(Cdh1) Inactivation Underlies the Point of No Return for Cell-Cycle Entry. Cell (2016) 166:167–80. 10.1016/j.cell.2016.05.077 PMC6649667 27368103

[100] Spencer SL Cappell SD Tsai FC Overton KW Wang CL Meyer T. The proliferation-quiescence decision is controlled by a bifurcation in CDK2 activity at mitotic exit. Cell (2013) 155:369–83. 10.1016/j.cell.2013.08.062 PMC4001917 24075009

[101] Asghar U Witkiewicz AK Turner NC Knudsen ES. The history and future of targeting cyclin-dependent kinases in cancer therapy. Nat Rev Drug Discov (2015) 14:130–46. 10.1038/nrd4504 PMC4480421 25633797

[102] Harbour JW Luo RX Dei Santi A Postigo AA Dean DC. Cdk phosphorylation triggers sequential intramolecular interactions that progressively block Rb functions as cells move through G1. Cell (1999) 98:859–69. 10.1016/S0092-8674(00)81519-6 10499802

[103] O’Leary B Finn RS Turner NC. Treating cancer with selective CDK4/6 inhibitors. Nat Rev Clin Oncol (2016) 13:417–30. 10.1038/nrclinonc.2016.26 27030077

[104] Gucalp A Proverbs-Singh TA Corben A Moynahan ME Patil S Boyle LA. Phase I/II trial of palbociclib in combination with bicalutamide for the treatment of androgen receptor (AR)+ metastatic breast cancer (MBC). Am Soc Clin Oncol (2016) 27(Supplement 6):VI81. 10.1093/annonc/mdw365.40

[105] Robinson TJ Liu JC Vizeacoumar F Sun T Maclean N Egan SE. RB1 status in triple negative breast cancer cells dictates response to radiation treatment and selective therapeutic drugs. PloS One (2013) 8:e78641. 10.1371/journal.pone.0078641 24265703 PMC3827056

[106] Trere D Brighenti E Donati G Ceccarelli C Santini D Taffurelli M. High prevalence of retinoblastoma protein loss in triple-negative breast cancers and its association with a good prognosis in patients treated with adjuvant chemotherapy. Ann Oncol (2009) 20:1818–23. 10.1093/annonc/mdp209 19556322

[107] Asghar U Herrera-Abreu MT Cutts R Babina I Pearson A Turner NC. Identification of subtypes of triple negative breast cancer (TNBC) that are sensitive to CDK4/6 inhibition. Am Soc Clin Oncol (2015) 33:15_suppl:11098. 10.1200/jco.2015.33.15_suppl.11098

[108] Liu CY Lau KY Hsu CC Chen JL Lee CH Huang TT. Combination of palbociclib with enzalutamide shows *in vitro* activity in RB proficient and androgen receptor positive triple negative breast cancer cells. PloS One (2017) 12:e0189007. 10.1371/journal.pone.0189007 29261702 PMC5737960

[109] Yeh S Miyamoto H Nishimura K Kang H Ludlow J Hsiao P. Retinoblastoma, a tumor suppressor, is a coactivator for the androgen receptor in human prostate cancer DU145 cells. Biochem Biophys Res Commun (1998) 248:361–7. 10.1006/bbrc.1998.8974 9675141

[110] Gao S Gao Y He HH Han D Han W Avery A. Androgen Receptor Tumor Suppressor Function Is Mediated by Recruitment of Retinoblastoma Protein. Cell Rep (2016) 17:966–76. 10.1016/j.celrep.2016.09.064 PMC5123835 27760327

[111] Thu KL Soria-Bretones I Mak TW Cescon DW. Targeting the cell cycle in breast cancer: towards the next phase. Cell Cycle (2018) 17:1871–85. 10.1080/15384101.2018.1502567 PMC6152498 30078354

